# Prescription precision: Evaluating antimicrobial prescription practices in paediatric and neonatal inpatients

**DOI:** 10.4102/sajid.v41i1.792

**Published:** 2026-02-16

**Authors:** Carla Theron, Maja van Aswegen, A’ishah da Costa, Heather Finlayson, Gugu Kali, Angela Dramowski

**Affiliations:** 1 Department of Paediatrics and Child Health, Faculty of Medicine and Health Sciences, Stellenbosch University, Cape Town, South Africa

**Keywords:** antimicrobial, antibiotic-resistance, prescription, neonate, paediatric, stewardship, prescription, PPS

## Abstract

**Background:**

There are limited data on antimicrobial prescribing quality in paediatric wards in African hospitals.

**Objectives:**

This study aimed to assess the quality of antimicrobial prescribing.

**Method:**

We conducted weekly point prevalence surveys (PPSs) (15 March 2025 – 05 April 2025), assessing antimicrobial use and prescribing quality in neonatal and paediatric medical wards at Tygerberg Hospital, Cape Town, South Africa. Inpatients with active antimicrobial prescriptions at 08:00 on PPS days were included, collecting data on demographics, antimicrobials, prescription quality and time to administration (hangtime) using electronic surveys.

**Results:**

We reviewed 842 prescription charts, of which 237 (28.1%) included antimicrobial agents (23.3% neonates vs 34.4% paediatric patients; *p* < 0.001). Antimicrobial use was highest in the neonatal intensive care unit (58.5%) and lowest in neonatal wards (20.0%). Most antimicrobial therapy was empiric (89.0%), with pneumonia (49.2%) leading in paediatric patients and sepsis (43.2%) in neonates. Ceftriaxone (20.9%) dominated paediatric use, while ampicillin (20.4%) and meropenem (16.7%) predominated in neonates. Overall, 49.8% of agents were ‘Access’, 49.3% ‘Watch’ and 0.9% ‘Reserve’ from the World Health Organization ‘AWaRe’ classification. The intravenous route of administration predominated (79.0%). Median treatment duration was 5 days (interquartile range [IQR] 5–7 days). In patients with sepsis, the median hangtime was 109 min (IQR 30 min – 205 min), with only 23.2% initiated within 60 min. Prescription quality was suboptimal: allergies were documented in 37.1%, infection source in 29.0% and stop dates in 38.0%.

**Conclusion:**

Antimicrobial prescribing in these neonatal and paediatric wards showed frequent use of broad-spectrum agents, delayed administration and incomplete documentation.

**Contribution:**

The findings highlight the need for improved stewardship practices to promote timely, appropriate and well-documented antimicrobial use.

## Introduction

Antimicrobial resistance (AMR) is a major threat to public health globally, particularly in low- and middle-income countries (LMICs), where the burden of AMR is greatest and access to new antimicrobials for the treatment of AMR infections is limited.^1^ The United Nations aims to reduce AMR-associated deaths by 10% by 2030 through antimicrobial stewardship (AMS) and surveillance initiatives.^2,3^ To support AMS surveillance efforts in LMICs, the World Health Organization (WHO) developed a standardised method to assess antimicrobial use with point prevalence surveys (PPS) and the AWaRe classification, which promotes the use of narrow-spectrum ‘Access’ antibiotics.^4,5^

Global PPS studies show regional differences in paediatric prescribing: Africa, Australia and Northern and Western Europe favour narrow-spectrum antibiotics, whereas Asia, Eastern and Southern Europe, North and Latin America use broader agents. Gentamicin and ampicillin dominate neonatal use worldwide, but prescribing quality is inconsistent, with indications often missing.^1^ The no-more-antibiotics and resistance (NO-MAS-R) global PPS found that 26% of neonates received antimicrobials, with higher use in LMICs (48%) compared to high-income countries (18%). Most were treated empirically for suspected sepsis.^6^

Few antimicrobial PPS have been conducted in South African children. One study across three academic hospitals reported 23% antimicrobial use, with 45% targeting healthcare-associated infections (HAI).^7^ A study of 18 public hospitals found 50% antimicrobial use, with 55% from the ‘Access’ group. Only 28% of prescriptions had microbial cultures requested, and just 39% of the culture results were documented.^8^ A cohort study at Tygerberg Hospital found that 92% of inpatients received antimicrobials, with third-generation cephalosporins and amoxicillin being the most frequently prescribed.^9^ Tygerberg Hospital’s neonatal unit has high rates of AMR in healthcare-associated bloodstream infections (HA-BSI), with 64% of *Klebsiella pneumoniae* being extended B-lactamase producers, 61% of *S. aureus* being methicillin-resistant and 72% of *Acinetobacter baumannii* being carbapenem-resistant.^10^

Globally there is a need to evaluate and improve the quality of antimicrobial prescribing, documentation and administration, focusing on aspects that influence the safety and effectiveness of antibiotic use. A key example of this is antimicrobial hangtime, as delays in antibiotic administration are linked to higher infection-associated mortality rates.^11^ At Tygerberg Hospital, a retrospective analysis of very low birth weight (VLBW, < 1500 g) neonates with bloodstream infection found that only 36% received their first antibiotic dose within 60 min of prescription.^12^

Given the current dearth of data on antimicrobial use and the quality of antibiotic prescribing in LMICs, we conducted repeated PPS of antimicrobial use in children admitted at a large South African hospital, describing prescribing patterns and key indicators of antimicrobial prescribing quality.

## Research methods and design

### Study setting

The study setting includes a tertiary academic hospital in Cape Town’s Eastern Metropole, which serves as a referral hospital for patients requiring specialist and subspecialist care from the Eastern Metropole as well as the West Coast, the Cape Winelands and the Overberg rural districts. Patients admitted to the paediatric medical and neonatal wards, including the neonatal and paediatric intensive care units and medical specialist and subspecialist wards, were eligible.

Common indications for hospital admission in the general paediatric wards are community-acquired infections, such as respiratory tract infections and acute gastroenteritis, and neonatal sepsis in the neonatal wards. Hospital-acquired infections are a major contributor to antimicrobial use in both the paediatric and neonatal wards and the intensive care units.^13^ A formal AMS programme was implemented at the hospital in 2016, incorporating weekly AMS ward rounds and a dedicated antimicrobial prescription chart.^14^ The antimicrobial prescription chart includes space to indicate the time of antibiotic prescription (to calculate hangtime) and has several other elements designed to enhance the quality of antimicrobial prescribing, such as reminders to review antibiotic prescriptions and document microscopy and culture sites and results. Ward stock antimicrobials vary between wards. In accordance with Tygerberg’s authorisation policy, only 72 h of unrestricted antimicrobials and authorised prescriptions may be dispensed at a time. Unauthorised prescriptions may receive only 24 h of doses while awaiting consultant approval. All ‘Reserve’ antibiotics and most ‘Watch’ antibiotics require authorisation by either a paediatric consultant or an infectious disease or microbiology specialist.

### Study design

A prospective, repeated PPS was performed with surveys conducted on the same weekday in four consecutive weeks (between 15 March 2025 and 05 April 2025). On each survey day, patient admissions were recorded, and antimicrobial prescription charts were reviewed alongside patients’ notes at the bedside. Inclusion criteria were patients admitted to the ward before 08:00 on the day of the survey to the paediatric or neonatal service, with an active prescription for antibiotics or antifungal agents, including single-dose antibiotics that were given or prescribed less than 24 h before the day of the survey. Antimicrobials stopped before 08:00 on the day of the survey were excluded, as well as antimicrobials initiated after 08:00 on the PPS day. Anti-tuberculosis and anti-retroviral agents were excluded. Patients in the wards who were already discharged prior to 08:00 on the day of the survey were excluded. Data were collected on the type of antimicrobials used, days of antimicrobials received, demographic characteristics of patients receiving antimicrobials, antibiotic hangtime (time from prescription to administration) and the proportion of charts completed correctly.

### Study definitions

An indication for antimicrobial therapy refers to the documented clinical condition or diagnosis for which the agent is prescribed. Hangtime is the elapsed time from when an antibiotic was written in the prescription to its actual administration to the patient (expressed in minutes). Duration of antimicrobial use was calculated based on the days of antimicrobial therapy received up to and including the survey day. Quality of antimicrobial prescriptions refers to the proportion of prescriptions that are correctly and legibly documented. These include patient information, antimicrobial dose, frequency, route and indication, and the prescriber’s signature, details and contact number.

### Data handling and analysis and ethical approval

Data were collected using an electronic survey using the REDCap platform. As part of the survey, we collected: (1) demographic data (age, weight, sex, primary diagnosis, indication for antibiotic use, type of therapy, place of acquisition), (2) antibiotic data (name of antimicrobial, duration prescribed, hangtime, route, availability of cultures), (3) quality indicators (how well prescribers were completing the antibiotic prescription) and (4) proportion of patients on antibiotics. Microsoft Excel was used to organise and analyse data. Descriptive statistics were used to summarise the collected data using Stata 19, with continuous variables summarised using averages and medians and interquartile range (IQR) for non-parametric data. Categorical variables were reported as frequencies and proportions, and comparisons were conducted using Chi-square tests as appropriate. A *p*-value < 0.05 was considered statistically significant.

### Ethical considerations

Ethical clearance to conduct this study was obtained from the Stellenbosch University’s Undergraduate Research Ethics Committee on 05 February 2025. The ethical clearance number is U25/01/367. A waiver for individual consent was approved by the hospital.

## Results

### Demographics

**FIGURE 1:**
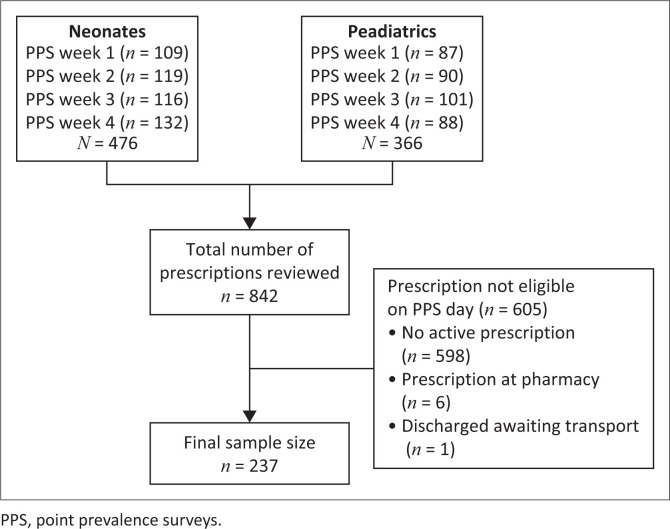
Determination of study population.

Over 4 PPS cycles, 842 infants (476 neonates and 366 paediatric patients) were admitted to the wards, of whom 237 (28.1%) were receiving antimicrobials on the day of the PPS, as noted in Figure 1. Neonates had a lower antimicrobial use rate compared to the paediatric inpatients (111/476 [23.3%] vs 126/366 [34.4%]; *p* < 0.001). The highest rate of antimicrobial consumption was observed in neonatal intensive care unit (NICU), and the lowest rate was in neonatal wards (24/41 [58.5%] vs 87/435 [20%]; *p* < 0.001). Antimicrobial consumption was 18/46 (39.1%) in the paediatric intensive care unit (PICU) and 108/320 (34%) in the paediatric wards. The median age of participants was 2 months (IQR 0.36–17 months) with a male predominance (*n* = 134, 56.5%). As shown in Table 1, most patients were treated empirically (*n* = 211, 89%). In paediatric patients, pneumonia was the leading indication (62/126; 49.2%), while in neonates, it was sepsis (48/111; 43.2%). Most infection episodes were community-acquired (124 [52.3%]).

**TABLE 1:** Demographic characteristics of paediatric and neonatal inpatients receiving antimicrobial therapy.

Variable	Total				Intensive care unit									Wards								
					NICU				PICU				*p*-value	Neonatal wards				Paediatric wards				*p*-value
	*n*	%	Median	IQR	*n*	%	Median	IQR	*n*	%	Median	IQR		*n*	%	Median	IQR	*n*	%	Median	IQR	
*N*	237	100.0	-	-	24	10.1	-	-	18	7.6	-	-	-	87	36.7	-	-	108	45.6	-	-	-
Age in months	-	-	2.0	0.4–17.0	-	-	0.4	0.1–1.3	-	-	4.0	2.0–6.0	< 0.001	-	-	0.3	0.1–0.9	-	-	18.0	5.0–60.0	< 0.001
Weight in kilograms	-	-	3.5	1.6–9.0	-	-	1.9	1.2–2.6	-	-	4.7	3.5–6.0	< 0.001	-	-	1.4	1.0–2.1	-	-	9.8	5.5–15.1	< 0.001
Sex (male)	134	56.5	-	-	13	54.2	-	-	10	55.5	-	-	-	57	65.5	-	-	54	50.0	-	-	-
**Indication for antibiotic(s)**																				
Pneumonia	74	31.2	-	-	4	16.7	-	-	12	66.6	-	-	0.001	8	9.2	-	-	50	46.3	-	-	< 0.001
Sepsis	58	24.5	-	-	9	37.5	-	-	2	11.1	-	-	0.056	39	44.8	-	-	8	7.4	-	-	< 0.001
Other†	42	17.7	-	-	5	20.8	-	-	4	22.2	-	-	0.914	12	13.8	-	-	21	19.4	-	-	0.295
Intra-abdominal infection	25	10.6	-	-	6	25.0	-	-	0	0.0	-	-	0.026	11	12.6	-	-	8	7.4	-	-	0.220
Meningitis	24	10.1	-	-	0	0.0	-	-	0	0.0	-	-	-	9	10.3	-	-	15	13.9	-	-	0.454
Congenital infection	8	3.4	-	-	0	0.0	-	-	0	0.0	-	-	0.001	8	9.2	-	-	0	0.0	-	-	< 0.001
Skin and soft tissue infection	6	2.5	-	-	0	0.0	-	-	0	0.0	-	-	-	0	0.0	-	-	6	5.6	-	-	0.026
**Infection acquisition**																				
Community acquired	124	52.3	-	-	3	12.5	-	-	7	38.9	-	-	0.047	36	41.4	-	-	78	72.2	-	-	< 0.001
Healthcare-associated	61	25.7	-	-	18	75.0	-	-	6	33.3	-	-	< 0.001	29	33.3	-	-	8	7.4	-	-	< 0.001
Unknown	52	22.0	-	-	3	12.5	-	-	5	27.8	-	-	0.212	22	25.9	-	-	22	20.4	-	-	0.414
Types of therapy																				
Definitive	26	11.0	-	-	2	8.3	-	-	3	16.7	-	-	0.409	14	16.1	-	-	7	6.5	-	-	0.031
Empirical	211	89.0	-	-	22	91.7	-	-	15	83.3	-	-	0.409	73	84.0	-	-	101	93.5	-	-	0.031

IQR, interquartile range; NICU, neonatal intensive care unit; PICU, paediatric intensive care unit.

† , Includes 26 (11%) with no indication, 6 (2.5%) urinary tract infections, 3 (1.3%) blocked ventriculo-peritoneal shunts, 2 (0.8%) Herpes infection, 1 (0.4%) upper respiratory tract infection, 1 (0.4%) severe acute malnutrition, 1 (0.4%) Kawasaki disease, 1 (0.4%) central line infection, 1 (0.4%) mediastinitis.

### Antimicrobial therapy

Of 237 prescription charts analysed, 382 individual antimicrobial agent prescriptions were active on the PPS days. Antibiotics were the most common antimicrobials prescribed (*n* = 339, 88.7%). Most antibiotics were prescribed intravenously (*n* = 302, 79.0%). Overall, the three most prescribed agents were meropenem (*n* = 48, 14.2%), ampicillin (*n* = 42, 12.4%) and ceftriaxone (*n* = 41, 12.1%). The most prescribed antibiotics in the paediatric population were ceftriaxone (*n* = 41, 20.9%) and amoxicillin/clavulanate (*n* = 19, 9.7%) compared to ampicillin (*n* = 38, 22.2%), gentamicin (*n* = 33, 19.3%) and meropenem (*n* = 31, 18.1%) in neonatal inpatients. As seen in Table 2, 49.8% (*n* = 169) of antibiotics prescribed were from the ‘Access’ group, 49.3% (*n* = 167) were from the ‘Watch’ group and 0.9% (*n* = 3) were from the ‘Reserve’ group. The neonatal wards had the highest usage of ‘Access’ antibiotics (67.9%).

**TABLE 2:** Antibiotics used according to World Health Organization AWaRe classification.

Variable	Total		Intensive care unit					Wards				
			NICU		PICU		*p*-value	Neonatal ward		Paediatric ward		*p*-value
	*n*	%	*n*	%	*n*	%		*n*	%	*n*	%	
*N*	339	100.0	37	10.9	31	9.1	-	134	39.5	137	40.5	-
**Access**	**169**	**49.8**	**10**	**27.0**	**10**	**32.2**	**0.637**	**91**	**67.9**	**58**	**42.3**	**< 0.001**
Ampicillin	42	12.4	4	10.8	1	3.2	0.366	34	25.4	3	2.2	< 0.001
Gentamicin	33	9.7	3	8.1	0	0.0	0.245	30	22.4	0	0.0	< 0.001
Amikacin	31	9.1	2	5.4	4	12.9	0.278	15	11.2	10	7.3	0.276
Amoxicillin-clavulanate	19	5.6	0	0.0	0	0.0	-	0	0.0	19	13.9	< 0.001
Amoxicillin	12	3.5	0	0.0	0	0.0	-	0	0.0	12	8.8	< 0.001
Benzyl penicillin	9	2.6	0	0.0	0	0.0	-	9	6.7	0	0.0	0.002
Co-trimoxazole	9	2.6	0	0.0	4	12.9	0.039	0	0.0	5	3.6	0.060
Cefalexin	5	1.5	0	0.0	0	0.0	-	2	1.5	3	2.2	1.000
Metronidazole	4	1.2	1	2.7	0	0.0	1.000	0	0.0	3	2.2	0.247
Cloxacillin	3	0.9	0	0.0	0	0.0	-	0	0.0	3	2.2	0.247
Chloramphenicol	1	0.3	0	0.0	0	0.0	-	1	0.7	0	0.0	0.494
Flucloxacillin	1	0.3	0	0.0	1	3.2	0.456	0	0.0	0	0.0	-
**Watch**	**167**	**49.3**	**27**	**73.0**	**21**	**67.8**	**0.637**	**43**	**32.1**	**76**	**55.5**	**< 0.001**
Meropenem	48	14.2	15	40.5	4	12.9	0.011	16	11.9	13	9.5	0.514
Ceftriaxone	41	12.1	0	0.0	9	29.0	< 0.001	0	0.0	32	23.4	< 0.001
Piperacillin-tazobactam	28	8.3	1	2.7	4	12.9	0.170	17	12.7	6	4.3	0.014
Vancomycin	16	4.7	10	27.0	1	3.2	0.009	0	0.0	5	3.6	0.060
Azithromycin	12	3.5	0	0.0	2	6.5	0.204	2	1.5	8	5.8	0.103
Cefotaxime	12	3.5	1	2.7	0	0.0	1.000	8	5.9	3	2.2	0.134
Ceftazidime	3	0.9	0	0.0	0	0.0	-	0	0.0	3	2.2	0.247
Ciprofloxacin	2	0.6	0	0.0	0	0.0	-	0	0.0	2	1.5	0.498
Levofloxacin	2	0.6	0	0.0	0	0.0	-	0	0.0	2	1.5	0.498
Cefixime	1	0.3	0	0.0	0	0.0	-	0	0.0	1	0.7	1.000
Erythromycin	1	0.3	0	0.0	1	3.2	0.456	0	0.0	0	0.0	-
Tobramycin	1	0.3	0	0.0	0	0.0	-	0	0.0	1	0.7	1.000
**Reserve**	**3**	**0.9**	**0**	**0.0**	**0**	**0.0**	-	**0**	**0.0**	**3**	**2.2**	**0.247**
Ceftazidime-avibactam	1	0.3	0	0.0	0	0.0	-	0	0.0	1	0.7	1.000
Colistin	1	0.3	0	0.0	0	0.0	-	0	0.0	1	0.7	1.000
Linezolid	1	0.3	0	0.0	0	0.0	-	0	0.0	1	0.7	1.000

NICU, neonatal intensive care unit; PICU, paediatric intensive care unit.

### Quality indicators

As shown in Table 3, of 237 antimicrobial prescription charts reviewed, patient details were documented on 98.3% (*n* = 233) of charts, and the ward name was recorded on 81.4% (*n* = 193). Patient weight was noted on 91.1% (*n* = 216) of charts, while allergies were documented on 37.1% (*n* = 88) of charts. Patient diagnoses were indicated on 29.1% (*n* = 69) of the charts, with 29.1% (*n* = 69) indicating the source of infection on the chart. Of individual antimicrobial prescriptions, 66.5% (*n* = 254) noted whether the drug was indicated as empiric or targeted therapy, with 71.2% (*n* = 272) noting the time of prescription.

**TABLE 3:** Quality indicators per chart and prescription.

Variable	Total		Intensive care units				Wards				*p*-value
			NICU		PICU		Neonatal wards		Paediatric wards		
	*n*	%	*n*	%	*n*	%	*n*	%	*n*	%
**Quality indicators per chart**											
*N*	237	100.0	24	10.1	18	7.6	87	36.7	108	45.6	-
Patient sticker or details	233	98.3	24	100.0	18	100.0	85	97.7	106	98.1	0.370
Ward name	193	81.4	22	91.7	16	88.9	67	77.0	88	81.5	0.327
Weight	216	91.1	22	91.7	18	100.0	76	87.4	100	92.6	0.310
Age or gestational age	75	31.6	11	45.8	3	16.7	18	20.7	43	39.8	0.007
Allergies	88	37.1	8	33.3	6	33.3	31	35.6	43	39.8	0.879
Active infection episode diagnosis	85	35.9	12	50.0	6	33.3	22	25.3	45	41.7	0.047
Active infection episode diagnosis source	69	29.1	12	50.0	6	33.3	22	25.3	29	26.9	0.106
STAT doses†	29	12.2	11	45.8	5	27.8	5	5.7	8	7.4	< 0.001
Microbiology cultures sent	85	35.9	13	54.2	6	33.3	33	37.9	33	30.6	0.170
**Quality indicators per antimicrobial prescribed**											
*N*	382	100.0	44	11.5	37	9.7	142	37.2	159	41.6	-
Drug indication (Empiric vs Targeted)	254	66.5	35	79.5	27	73.0	85	59.9	107	67.3	0.075
Start date	380	99.5	44	100.0	37	100.0	142	100.0	157	98.7	0.690
Stop date	145	38.0	14	31.8	14	37.8	72	50.7	45	28.3	0.001
Time of prescription	271	71.1	38	86.4	17	45.9	117	82.4	99	62.7	< 0.001
Signature and name legibility	322	84.3	40	90.9	28	75.7	121	85.2	133	83.6	0.313
Contact details of prescriber	98	25.7	14	31.8	3	8.1	45	31.7	36	22.6	0.011
Time of dosing all legible	371	97.1	43	97.7	37	100.0	142	100.0	149	93.7	0.006
**Days on therapy**											
*N*	382	100.0	44	11.5	37	9.7	142	37.2	159	41.6	-
< 3 days	238	62.3	19	43.2	21	56.8	99	69.7	99	62.3	0.014
4–6 days	100	26.2	22	50.0	11	29.7	29	20.4	38	23.9	0.001
≥ 7 days	44	11.5	3	6.8	5	13.5	14	9.9	22	13.8	0.535

NICU, neonatal intensive care unit; PICU, paediatric intensive care unit.

† , Derived from the Latin word *statim*, meaning given immediately.

**FIGURE 2:**
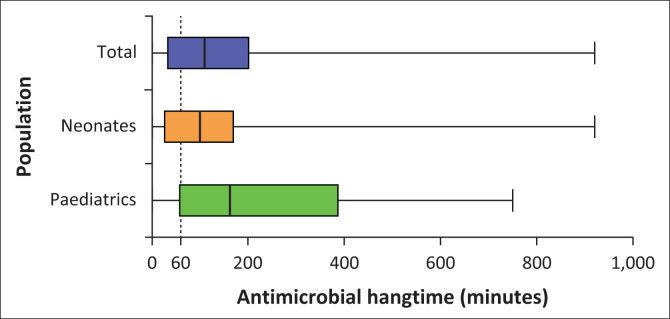
Antimicrobial hangtime.

The start date was indicated in 99.5% (*n* = 380), but only 38.0% (*n* = 145) had a stop date present. On the day of the PPS, most patients, 62.3% (*n* = 238), had received less than 3 days of therapy. A further 26.2% (*n* = 100) had been ongoing for 4–6 days, while 11.5% (*n* = 44) had been prescribed for 7 days or more, as seen in Table 3. The hangtime could be calculated in 67.0% (*n* = 256) of cases. In cases of sepsis (*n* = 58, 24.5%), the proportion of antimicrobial prescriptions with a hangtime of less than or equal to 60 min was 23.2% (*n* = 80), with a median hangtime of 109 min (IQR 30 min – 204.5 min), as noted in Figure 2. In the neonatal cohort, the median hangtime was 100 min (IQR 25 min – 172.5 min), with 42.2% receiving their antibiotics within 60 min. In the paediatric cohort, the median hangtime was 162.5 min (IQR 55 min – 390 min), with 31.3% receiving their antibiotics within 60 min.

The most frequently omitted component of the antimicrobial prescription chart was information regarding the collection of specimens for microscopy, culture and sensitivity (specimen type, date and site) (*n* = 3; 1.3%). Similarly, pathogen identification and sensitivity results were rarely documented, appearing on only 0.8% (*n* = 2). However, 35.9% (*n* = 85) of prescription charts did include information on whether microbiology cultures had been submitted or not.

## Discussion

This study assessed antimicrobial use and prescribing quality in paediatric and neonatal wards and ICUs. A third of patients admitted were receiving antimicrobials, with most prescriptions being empiric and targeting community-acquired infections, with an equal distribution in the use of ‘Access’ and ‘Watch’ antibiotics. Only 25% of patients with sepsis received antibiotics within the recommended golden hour, and documentation of key prescribing elements, such as the indication, microscopy results and prescriber details, was often poorly completed. These findings highlight the need for strengthened AMS efforts aimed at improving the quality of antimicrobial use.

Overall, the antimicrobial utilisation rate (28.1%) was lower than that found in the Antibiotic Resistance and Prescribing in European Children (ARPEC) study (36.7%) but higher compared to a PPS conducted in three South African hospitals (22.9%).^1,7^ The NICU’s antimicrobial utilisation (58.5%) was considerably higher compared to other LMICs (26% – 48%) in the NO-MAS-R PPS study but lower than the 93% in another multicentre PPS.^6,15^ The higher antimicrobial use in our NICU likely reflects the high clinical severity of illness and impaired IPC strategies, often because of overcrowding and a high patient-to-healthcare-worker ratio compared to patients hospitalised in other wards.

Pneumonia was the leading indication for antimicrobial prescription in the paediatric cohort, which is consistent with national data, indicating respiratory tract infections as the leading indication for hospitalisation and death in children under 5 years. Numbers have declined substantially following the introduction of the pneumococcal conjugate vaccine in the childhood immunisation schedule.^16^ Sepsis was the most common indication for antimicrobial use in neonates, in keeping with the NO-MAS-R global PPS and another PPS done in Nigeria.^6,17^ Sepsis is a large contributor to global morbidity and mortality, especially in neonates in LMICs.^18,19^ Healthcare-associated infections contributed to 25.7% of infections, which is lower than the 45.6% prevalence reported in other South African hospitals.^7^ However, several prescription charts lacked information regarding place of infection acquisition, which may have misrepresented these infection rates.

The three most commonly prescribed agents were meropenem, ampicillin and ceftriaxone. Ceftriaxone was the most prescribed antimicrobial in the paediatric population, consistent with a 2018 cohort study at the same facility. Ceftriaxone is the first-line therapy for paediatric pneumonia at Tygerberg Hospital. This was implemented in 2023 after the release of the new Standard Treatment Guidelines. Ceftriaxone has a similar spectrum of antibiotic cover, is more affordable and is easier to administer when compared to amoxicillin-clavulanate, which is the first-line agent according to the 2023 South African Standard Treatment Guidelines.^20^ This also explains the high use of empiric antimicrobials (93.5%) in the paediatric wards, as pneumonia’s low culture yield limits the use of targeted therapy.^21^ Ampicillin and gentamicin were the most prescribed antibiotics in the neonatal population, consistent with the WHO-recommended empiric antibiotic regimen for suspected early-onset neonatal sepsis (EONS).^19^ Presumed sepsis in neonates is often managed empirically, because of the non-specific clinical presentation.^19,22^ The NICU had a substantially higher rate of empirical use (91.7%) compared to the 55% reported in the NO-MAS-R global PPS, although the study does not present separate data for high-income countries and LMICs, limiting direct comparison with our findings.^6^ Most ward neonates received less than 3 days of empiric therapy, reflecting compliance with the NICE 2021 guidelines.^22^ Recent data suggest high rates of bug–drug mismatch for this EONS regimen in some African neonatal units, owing to changes in pathogen spectrum and AMR rates.^10,23^ Meropenem (40.5%) and vancomycin (27.0%) were the most used agents in the NICU. Meropenem with or without vancomycin is empirically prescribed for neonates admitted to the ICU with HAI.

Based on the WHO AWaRe classification, 49.8% of antimicrobials were from the ‘Access’, 49.3% from the ‘Watch’ and 0.9% from the ‘Reserve’ categories. This is similar to a 2021 study conducted in South Africa, which found 48% of antibiotics prescribed in tertiary hospitals were from the ‘Access’ group.^8^ The lower use of ‘Access’ antibiotics may reflect the more complicated patient profile, with longer patient stays referred to this tertiary centre. Antibiotic prescribing in the neonatal wards nearly meets the WHO AWaRe target, with about 70% of prescriptions falling into the ‘Access’ category.^24^ The NICU had the largest proportion of patients receiving antibiotics from the ‘Watch’ category at 73.0%. This is substantially higher than findings from other African NICUs reported in the NO-MAS-R study.^6^ The trend likely reflects institutional guidelines endorsing use of ‘Watch’ agents (piperacillin–tazobactam, amikacin or meropenem) as empiric therapy for suspected healthcare-associated bloodstream infections (HA-BSI), considering the high rates of AMR in HA-BSI in our neonatal unit.^10^

Sepsis was diagnosed in 58 (24.5%) cases. Within this group, 112 antimicrobials were prescribed. The hangtime was only calculable in 71.4% (*n* = 80). Of that, only 23.2% (*n* = 26) of cases had a hangtime of less than or equal to 60 min. The surviving sepsis campaign recommends the administration of empiric antibiotics within the first hour of clinical diagnosis, with the goal of shortening time to microbiological clearance of the bloodstream and decreasing mortality.^25,26^ A previous study at a similar institution reported that 37% of VLBW neonates received antimicrobials within the first hour, which is lower than the 42.2% found in neonates in this study.^12^ However, incomplete records limit the interpretation. In the paediatric cohort, only 31.3% of prescribed antibiotics were given in the ideal 60 min timeframe. This figure does not consider that many children with sepsis will be referrals from peripheral hospitals or the labour ward, where they may have received antibiotics. The hospital’s paper-based system limits accurate monitoring; electronic prescribing could improve measurement. The underutilisation of ‘stat’ dosing contributed to delays, as shown in a US initiative where timely administration of antimicrobials improved from 33% to 77% with appropriate use of ‘stat’ dosages.^27^

Most prescriptions had relevant patient details, ward name and patient weight on the front page. Paediatric dosing is weight-based; thus, it is crucial to have this information clearly documented. The NICU showed the highest rate in documenting age/gestational age, diagnosis, infection source and stat dosages. However, overall completion rates were poor. The difference is likely because of the NICU’s lower total patient load and higher level of care, allowing more attention to chart completion. Diagnosis, source and microscopy results in the prescription chart were poorly completed, limiting AMS efforts. This may not necessarily reflect the information available in the medical record. A retrospective review at a Johannesburg hospital found poor chart completion but still found a 7% reduction in antimicrobial prescriptions after introducing a new antimicrobial prescription chart.^28^

For each prescribed agent, more than two-thirds of cases had been completed, whether the indication for the agent was empiric or definitive. Although almost all cases had antimicrobial start dates completed, only 37.9% had stop dates indicated. Completion of antimicrobial stop dates is essential for stewardship to avoid prolonged treatment courses and for prompting clinicians to consider an IV-to-oral switch. The contact details of the prescriber were missing in almost 75% of cases, making communication between pharmacist and clinicians difficult.

One of the major limitations of this study was that it only considered patients from one tertiary hospital, which may have different prescribing patterns than hospitals in the periphery. The PPS method assesses data at a single point in time and may not reflect prescribing practices over time; for example, empiric management may become targeted management once cultures become available. The short timeframe could skew data because of seasonal variations in disease. Clinical outcomes were not included in this study. Because microbiological results were not properly indicated on the antimicrobial prescriptions, it was not possible to collect that data.

This study will contribute to the AMS efforts at this hospital and provide invaluable insight into the ways in which prescribing can be improved. Additional prescriber training should be provided to make them aware of common errors and pitfalls to improve the overall quality of prescribing. Nursing staff in charge of giving medications, especially in acute settings (such as emergency and the ICU), should be informed of ‘stat’ antimicrobial orders when they are written up, to increase the proportion of patients who receive their antimicrobial within 60 min. It would be valuable to repeat a similar study after quality improvement initiatives have been put in place to assess the success thereof.

## Conclusion

This study provides a comprehensive evaluation of antimicrobial prescribing practices in paediatric and neonatal wards. There was a high prevalence of empiric therapy with frequent use of ‘Watch’ and ‘Reserve’ antimicrobials and poor completion of antimicrobial charts. Fewer than half of the prescribed agents were given within the ideal 60 min timeframe. These findings highlight the need to strengthen AMS through enhanced managerial oversight and expanded AMS activities in each ward, with all team members actively engaged in stewardship and accurate chart completion. Future research should focus on the implementation and impact of quality improvement initiatives on prescribing behaviour, AMR patterns and clinical outcomes in paediatric populations in resource-limited settings.
